# Meningococcal C Disease Outbreak Caused by Multidrug-Resistant *Neisseria meningitidis*, Fiji

**DOI:** 10.3201/eid3101.240476

**Published:** 2025-01

**Authors:** Aneley Getahun Strobel, Aalisha Sahukhan, Anaseini Ratu, Jimaima Kailawadoko, Isireli Koroituku, Shalini Singh, Samuel McEwen, Sakiusa Baleivanualala, Mathilda Wilmot, Silivia Matanitobua, Kerrie Stevens, Anaseini Vesikula, Talica Cabemaiwai, Raquel Cooper, Mere Taufa, Jokaveti Tadrau, Kristy Horan, Daniel Faktaufon, Benjamin P. Howden, Eric Rafai

**Affiliations:** The University of Melbourne at Peter Doherty Institute for infection and Immunity, Melbourne, Victoria, Australia (A.S. Getahun, M. Wilmot, K. Stevens, R. Cooper, K. Horan, B.P. Howden); Fiji National University, Suva, Fiji (A.S. Getahun, A. Ratu); Fiji Ministry of Health and Medical Services, Suva (A. Sahukhan, J. Kailawadoko, I. Koroituku, S. Singh, S. McEwen, S. Baleivanualala, S. Matanitobua, A. Vesikula, T. Cabemaiwai, M. Taufa, J. Tadrau, D. Faktaufon, E. Rafai); Centre for Pathogen Genomics, The University of Melbourne, Melbourne (B.P. Howden)

**Keywords:** Bacteria, *Neisseria meningitidis*, meningococcal C, meningitis/encephalitis, drug resistance, antimicrobial resistance, epidemiology, meningococcus, Oceania, outbreaks, vaccination, Fiji

## Abstract

We describe an outbreak of invasive meningococcal disease (IMD) caused by *Neisseria meningitidis* serogroup C in Fiji. We created surveillance case definitions and collected data by using standard investigation forms. Bacterial identification, antimicrobial susceptibility testing, and PCR were performed in Fiji. Molecular testing was conducted at the Microbiological Diagnostic Unit in Melbourne, Victoria, Australia. During January 2016–December 2018, a total of 96 confirmed or probable IMD cases were reported. Of case-patients, 61.5% (59/96) were male and 38.5% (37) female, 84.4% (81) were indigenous people of Fiji, and 70.8% (68) were children <15 years of age. Annual incidence increased from 1.8/100,000 population in 2016 to 5.2/100,000 population in 2018. Serogroup C multilocus serotype 4821 that is resistant to ciprofloxacin was prevalent (62.1%, 41/66). Public health measures, which included targeted mass vaccination with monovalent meningitis C vaccine, were effective in controlling the outbreak. We observed a rapid decline in meningitis C cases in subsequent years.

Invasive meningococcal disease (IMD) is caused by the gram-negative bacterium *Neisseria meningitidis*. Infection cause invasive and life-threatening infection including meningitis and meningococcaemia (*1*,*2*). IMD is caused by bloodstream invasion of 1 of 6 virulent serogroups, A, B, C, X, Y, or W (*1*). Global distribution of IMD varies; however, the disease burden in Pacific Island countries is not well documented. Studies from Australia and New Zealand reported endemic and epidemic trends caused by meningococcal serogroups B (MenB) and C (MenC) in the 1990s (*3*,*4*) but a major reduction in IMD cases after the introduction of routine vaccination (*5*,*6*).

In 2018, an outbreak of IMD occurred in Fiji, which is a small island developing state with a population of 884,887 as of 2017 (*7*). Health services are administrated by the Ministry of Health and Medical Services (MoHMS), which is split into 4 divisions: Central, Western, Northern, and Eastern. Each division is further separated into subdivisions, medical areas, and zones. There are 3 divisional hospitals (Colonial War Memorial, Lautoka, and Labasa) with microbiological culture capabilities. Primary healthcare is provided through 19 subdivisional hospitals and 189 peripheral facilities (*8*). A limited number of private facilities exist, largely in urban centers. Within the MoHMS is the Fiji Centre for Disease Control (Fiji CDC), which manages national infectious disease surveillance and includes the National Public Health Laboratory (NPHL). National IMD surveillance is conducted through 3 systems: the National Notifiable Disease Surveillance System, which reports clinical or culture confirmed cases (*9*); the Vaccine Preventable Disease surveillance (VPD), which collects information on laboratory confirmed cases (culture, antigen test, and PCR) of IMD with serogroup data (*9*); and the Early Warning, Alert and Response Surveillance system, which reports clinically suspected meningitis cases (*10*).

IMD was previously uncommon in Fiji, which reported 5–10 cases annually during 2007–2015, according to the MoHMS-Health Information Unit. Meningococcal vaccination is not in the national immunization schedule, and literature on the epidemiology of IMD is scarce. During 2004–2016, the effect of meningococcal meningitis was evaluated in 2 studies that were part of a larger study set on meningitis in pediatric and adult populations; of note, all strains were MenB (*11*,*12*). Similarly, serogroup data from National VPD surveillance recorded the occurrence of MenB and serogroup W (MenW) in Fiji but no record of MenC before 2016. Beginning in October 2016, the number of IMD cases has increased steadily. By 2017, an institutional outbreak had occurred, and by early 2018, a national outbreak was declared. The confirmed prevalent IMD was MenC and multilocus sequence type (MLST) 4821. In this article, we describe the epidemiology of IMD in Fiji during 2016–2018 with a focus on the 2017–2018 MenC outbreak, the interventions used for outbreak control, and postoutbreak surveillance.

## Methods

### Case Definition

Surveillance case definitions were adopted from the World Health Organization (*13*) and Centers for Disease Control and Prevention (*14*). We defined a suspected case as illness in a patient with a sudden onset of fever and >1 of the following: severe headache, nausea and vomiting, neck stiffness, altered consciousness, or skin rashes such as petechiae or purpura. We defined a probable case as a suspected case with an epidemiologic link (living in the same house or dormitory, attending same school, class, or daycare, sharing food or drink, or direct exposure to the case’s oral secretions) to a confirmed case. We defined a confirmed case as a suspected or probable case with >1 of the following: isolation of *N. meningitidis* from cerebral spinal fluid (CSF) or blood, positive PCR, or positive direct antigen test.

We defined an outbreak as a substantial increase in IMD within a defined population above what is expected for that place and time (*13*). We defined an institutional outbreak as >2 cases of IMD (probable or confirmed cases) within 4 weeks among persons with a common institution-based affiliation (such as attending the same school) but no close contact with each other in a grouping that makes epidemiologic sense (*14*).

### Data Collection

We used a standard case investigation form to capture demographic (age, sex, ethnicity, and residential location), clinical (signs and symptoms, laboratory results, clinical outcome), and epidemiologic (date of onset, contacts, etc.) variables. We collected data through review of medical folders, laboratory reports, and interviews of patients and family members.

### Laboratory Procedures

*N. meningitidis* was identified by using approved protocols at the 3 divisional laboratories in Fiji. We determined the *N. meningitidis* serogroups (A, C, Y, and W) were determined by using Wellcogen Bacterial Antigen Rapid Latex Agglutination Test (Thermo Scientific, https://www.thermofisher.com).

Antimicrobial susceptibility testing was conducted by using the disc diffusion method in accordance with the Clinical and Laboratory Standards Institute guidelines (*15*). We sent all confirmed isolates to NPHL and also referred all CSF samples from patients meeting case definitions to NPHL for real-time PCR analysis. We extracted *N. meningitidis* DNA by using QIAamp DNA mini kit (QIAGEN, https://www.qiagen.com) according to manufacturer instructions. We conducted sodC gene–based real-time PCR by using singleplex assays with primers and probes specific for *N. meningitidis* (Appendix 1 Table 1). We prepared the reaction mix to a final volume of 25 μL that included 2 μL of each primer (forward and reverse) and probe, 4.5 μL nuclease free water, 12.5 μL of iTaq mastermix (Bio-Rad Laboratories, https://www.bio-rad.com), and 2 μl DNA template. We then loaded the final volume onto the CFX96 analyzer (Bio-Rad) with thermocycling conditions of 1 cycle of 95°C for 3 minutes, followed by 39 cycles of 95°C for 5 seconds and 60°C for 5 seconds. We sent 29 samples (5 from 2017 and 24 from 2018) to the World Health Organization regional reference laboratories for invasive bacterial VPD at the Microbiological Diagnostic Unit public health laboratory (MDU) in Melbourne, Victoria, Australia, for serogrouping and whole-genome sequencing (*16*).

We used the Nextera XT DNA Library Preparation Kit (Illumina, https://www.illumina.com) to generate libraries that we then sequenced on the Nextseq 500 and NextSeq 550 (Illumina) platforms. We generated assemblies by using SPAdes v3.15.2 (https://github.com/ablab/spades) (*17*) and identified antimicrobial resistance genes and mutations by using abritAMR v1.0.10 with the “–species Neisseria” flag (https://github.com/MDU-PHL/abritamr) (*18*). We further examined the *penA* allele by a comparison of assemblies with *penA* alleles in PubMLST (https://pubmlst.org). We identified the *N. meningitidis* serogroup, Bexsero antigen sequence type, and the FetA protein type in silico by using meningotype (https://github.com/MDU-PHL/meningotype). We identified the MLST by using *in silico* sequence typing (https://github.com/tseemann/mlst). We considered how the sequences fit into the global context of *N. meningitidis* on the basis of raw read data availability and membership in the ST4821 clonal complex (CC). We generated a phylogenetic tree by using the bohra genomics pipeline (https://github.com/MDU-PHL/bohra), which implements snippy version 4.4.5 (https://github.com/tseemann/snippy) and IQtree version 2.0 (http://www.iqtree.org) by using the NM_FAM18 reference. We conducted recombination correction by using gubbins (https://github.com/nickjcroucher/gubbins); however, there was no discernible effect on the genomic relationships in this dataset. 

To assess the relationships of the Fiji strains within the global context of ST4821 CC, we downloaded all available *N. meningitidis* ST4821 CC assemblies from PubMLST on August 1, 2024. We generated a neighbor-joining tree of that data by using mashtree (*19*). We generated all tree figures by using R version 4.3.1 (The R Project for Statistical Computing, https://www.R-project.org) and R Studio version 2023.09.1+494 (Posit, http://www.rstudio.com) with the following library packages; ggtree version 3.10.0 (*20*), ggplot2 version 3.4.4 (*21*), phytools version 2.0.3 (*22*), and ggnewscale version 0.4.9 (https://CRAN.R-project.org/package=ggnewscale).

### Data Analysis

We conducted data analysis by using Excel (Microsoft, https://www.microsoft.com) and SPSS Statistics 25 (IBM, https://www.ibm.com). We created an incidence map by using EpiInfo 7.2. (https://www.cdc.gov/epiinfo). We characterized demographics, clinical profiles, *N. meningitidis* serogroup patterns, and patient outcomes by using descriptive analysis. We drew the epidemic curve from actual (n = 87) or estimated date of onset (n = 9) by subtracting the incubation period from the date of first visit or date of hospital admission. We used census data from 2017 for overall and age-sex–specific incidence calculation (*7*). We calculated incidence by medical division and subdivision by using the 2015 population estimates from MoHMS because census data cannot be disaggregated for medical subdivisions (*23*). We calculated overall and specific case-fatality rate (CFR) for age and gender groups.

## Results

### Demographic Characteristics

During January 2016–December 2018, a total of 96 cases of IMD were reported in Fiji. Of those, 82 cases (85.4%) were confirmed, and 14 cases (14.6%) were probable. Most reported case-patientss were male (61.5%, n = 59), iTaukei (indigenous people of Fiji; 84.4%, n = 81), and children <15 years of age (71.8%, n = 68) (Table 1). The median age was 9.5 years (interquartile range 2–14 years). Approximately half of cases (53.1%, n = 51) were reported from the Central division.

**Table 1:** Demographic characteristics of confirmed and probable cases in meningococcal C disease outbreak caused by multidrug resistant *Neisseria meningitidis*, Fiji, 2016–2018*

Characteristic	No./total no. (%)
Sex	
M	59/96 (61.5)
F	37/96 (38.5)
Ethnicity	
iTaukei, indigenous people of Fiji	81/96 (84.4)
Indian descent from Fiji	7/96 (7.3)
Other ethnicity from Fiji	5/96 (5.2)
Non-Fiji citizen	3/96 (3.1)
Age group, y	
<1	18/96 (18.8)
1–4	14596 (15.6)
5–9	15/96 (15.6)
10–14	20/96 (21.8)
15–19	17/96 (17.7)
>20	11/96 (11.5)
Medical division	
Central	51/96 (53.1)
Western	29/96 (30.2)
Eastern	9/96 (9.4)
Northern	7/96 (7.3)
Method of confirmation	
Blood or CSF culture	34/82 (41.5)
CSF PCR	24/82 (29.3)
Blood or CSF culture and CSF PCR	14/82 (17.1)
CSF positive antigen test	9/82 (11.0)
Postmortem PCR	1/82 (1.2)

*CSF, cerebrospinal fluid.

**Figure 1:**
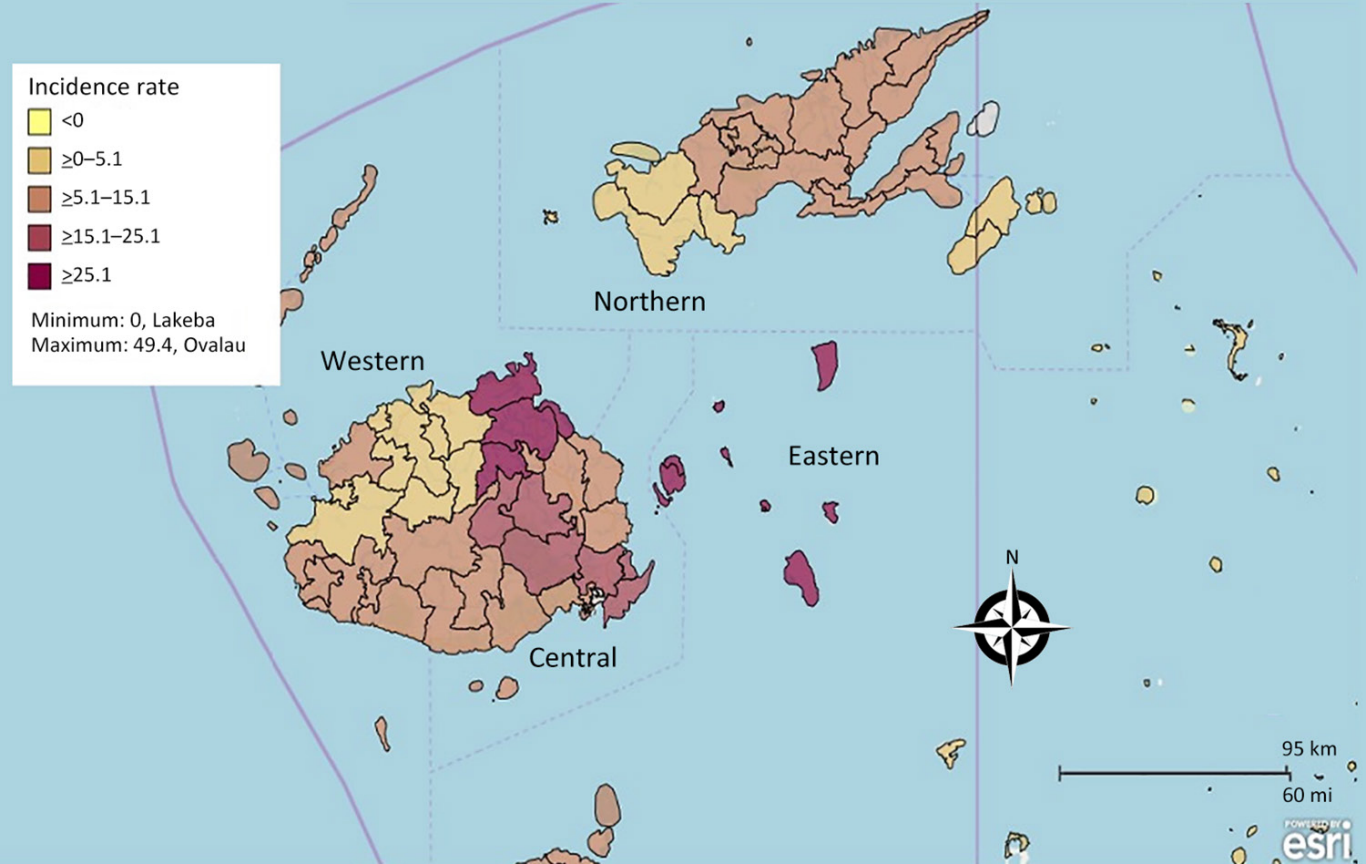
Cumulative incidence (cases/100,000 population) of confirmed and probable invasive meningococcal disease cases in Fiji, by medical subdivision and medical areas, 2016–2018.

Annual incidence of IMD tripled from 1.8/100,000 population (confirmed cases) in 2016 to 5.2/100,000 population in 2018 (confirmed and probable cases). The cumulative incidence for the period was 10.8 cases/100,000 population. The cumulative incidence was highest among children <5 years of age (35.9 /100,000 population) and men (13.2/100,000 population). The highest calculated cumulative incidence of 46.6/100,000 population was for male children <5 years of age. The Eastern division had the highest cumulative incidence of 23/100,000 population, followed by the Central division at 13/100,000 population (Figure 1).

There were 15 deaths from IMD during the period, for an overall CFR of 14.6%. The CFR was 2× higher in women (21.6%) than in men (10.2%) (Appendix 1 Figure).

### Outbreak Detection and Spread

**Figure 2:**
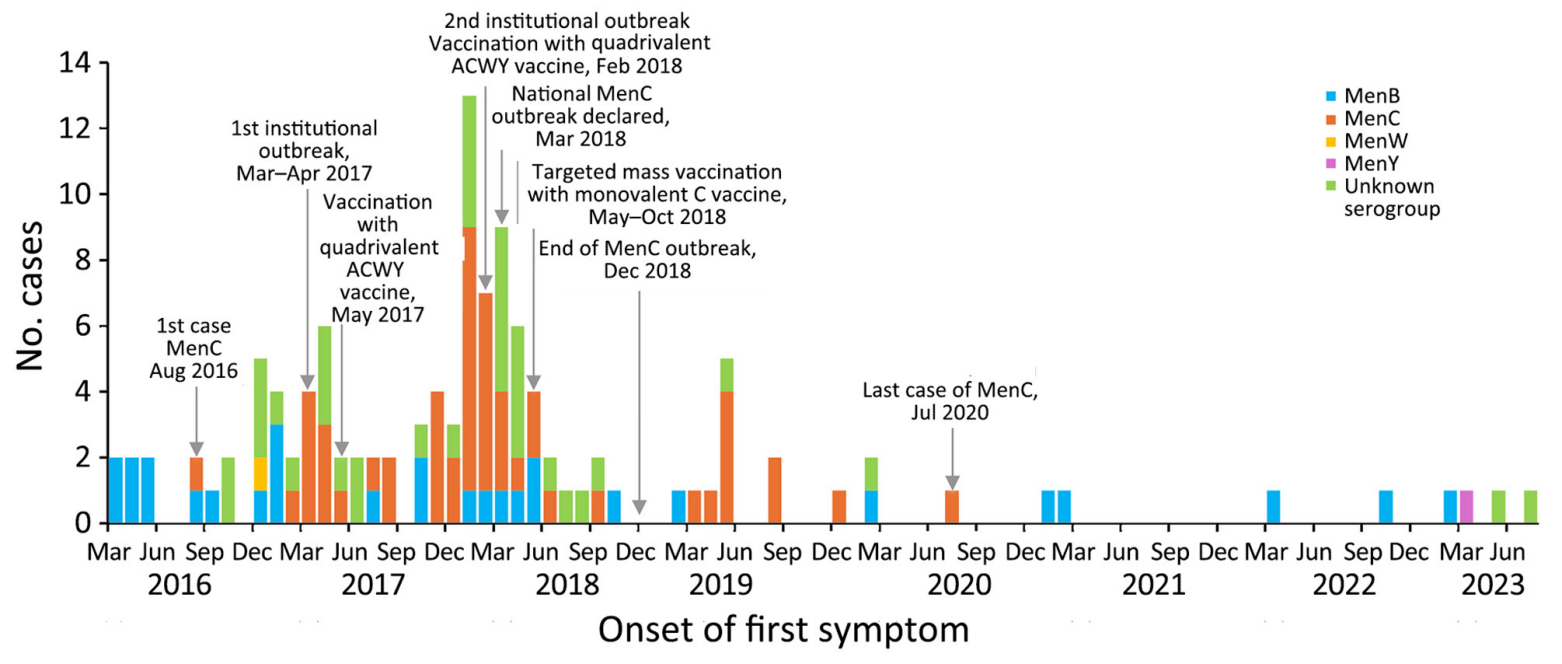
Epidemic curve showing the number of confirmed and probable invasive meningococcal disease cases in Fiji reported monthly by serogroup, January 2016–August 2023. Surveillance was interrupted in 2021 because of the COVID-19 outbreak. MenB, *Neisseria meningitidis* serogroup B; MenC, *N. meningitidis* serogroup C; MenW, *N. meningitidis* serogroup W; MenY, *N. meningitidis* serogroup Y.

In the fourth quarter (October–December) of 2016, there was a progressive increase in reported cases of IMD (n = 7), and most (n = 5) had an unspecified serogroup. Initially, most cases were from the Central (n = 5) and Western divisions (n = 2). In the first quarter of 2017, there were 3 IMD cases reported from a boarding school in the Eastern division. By May 2017, there were 2 additional cases reported from the same school, including 1 death. Of those 5 cases, 4 samples were positive for MenC, MLST 4821, resistant to ciprofloxacin and penicillin. The occurrence of 3 confirmed cases in 3 consecutive weeks at the boarding school resulted in the declaration of an institutional MenC outbreak. By the second quarter of 2017, community cases of IMD continued to rise, and cases were reported from all 4 medical divisions. MenC activity increased and became the predominant strain (Figure 2).

Case numbers of IMD dropped in the third quarter of 2017 but peaked in the first quarter of 2018, when 29 cases were reported (Figure 2). In February 2018, a second institutional outbreak was reported in a boarding school in the Western Division. A total of 3 confirmed and 8 probable cases of MenC were reported from the boarding school. A national outbreak was declared on March 20, 2018, and widespread public health interventions were implemented in the country, including targeted mass vaccination (*24*). During 2018, the number of cases progressively declined, and the outbreak was officially declared over in the first week of December 2018 (*25*).

### Clinical Features

The median time from symptom onset to seeking care at a health facility was 1 day (interquartile range 1–2 days). Common symptoms included fever (97.8%, n = 88), vomiting (61.1%, n = 55), headache (47.8%, n = 43), and rashes (31.1%, n = 28) (Appendix 1 Table 2).

### Laboratory Findings

A total of 73 CSF samples were available for culture and PCR testing. The positivity rate for CSF PCR was 52.1% (n = 38) and for culture was 19.2% (n = 14). *N. meningitidis* grew in 51.9% (41/79) of blood cultures. We determined the serogroup of 66 isolates and found 62.1% (n = 41) were MenC, 33.3% (n = 22) MenB, and 1.5% (n = 1) MenW; 3.1% (n = 2) were inconclusive. The first case of IMD caused by MenC was reported in August 2016. All MenC strains identified in 2017 and 2018 were MLST 4821.

According to the antimicrobial susceptibility testing results from the laboratories in Fiji, 35% (n = 15) of the total and 46% (n = 11) of the MenC strains were susceptible to penicillin (Appendix 1 Tables 3, 4). Susceptibility to ciprofloxacin was 77% (n = 30) in total for all strains and 67% (n = 14) for the MenC strains. However, all the MenC isolates (n = 15) sent to MDU were reported as decreased susceptibility or resistant to ciprofloxacin.

**Figure 3:**
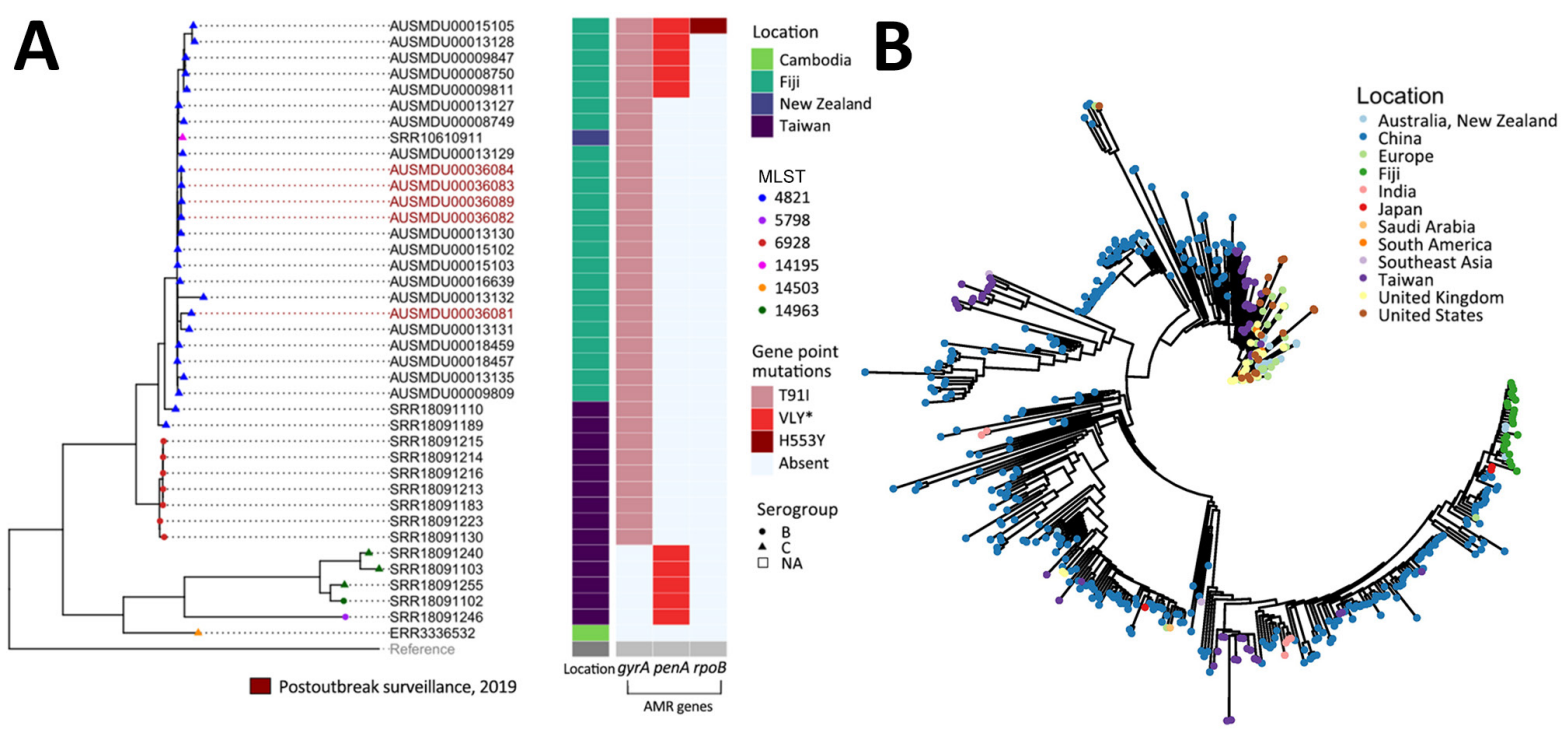
Genomic relatedness between *Neisseria meningitidis* strains identified from invasive meningococcal disease outbreak in Fiji, January 2016–August 2023, and publicly available gene sequences. A) Phylogenetic tree of Fiji MLST 4821 isolates and related sequences. Bar charts indicate location and presence or absence of antimicrobial resistance genes. A total of 18 MenC strains associated with the outbreak and 5 MenC strains from the post outbreak surveillance period were typed as MLST 4821 and included in analysis. All 23 strains were found to contain the *gyrA* point mutation T91I, and only 1 strain contained the *rpoB* point mutation H553Y. Of the 18 strains associated with the outbreak, 5 contained the *penA* point mutations; A510V, F504L, and N512Y. B) Mashtree generated neighbor-joining tree of publicly available PubMLST (https://pubmlst.org) data typed as MLST 4821 clonal complex and the MLST 4821 Fiji strains. When placed in this global context, the strains from Fiji form a separate cluster and appear to have a common ancestor with strains that have been isolated in China. AMR, antimicrobial reistant; MenC, *N. meningitidis* serotype C; MLST, multilocus sequence type; NA, not available.

Of the MLST4821 CC sequences available, most were serogroup C, MLST 6928 or MLST 5798, except 1 strain from MLST 14963 that identified as MenB (*26*). We observed a high level of genomic relatedness among the MLST 4821 strains from Fiji, which appear to share a common ancestor (Figure 3, panel A). Of interest, 1 strain within this cluster typed as MLST 14195 and was isolated from New Zealand. When placed in a global context, the Fiji strains form a separate cluster from any other global isolates available on PubMLST and appear to have a common ancestor from strains isolated in China (Figure 3, panel B).

The sequences from MLST4821 (Fiji), MLST14195 (New Zealand), and MLST6928 (Taiwan) all carry the same *gyrA* mutation (T91I) that is not present in the other sequences. Of note, only 1 Fiji isolate had an *rpoB* gene mutation (Figure 3, panel A). Of interest, the *penA* mutations (A510V, F504L, and N512Y) identified in 5 of the sequences from Fiji and 5 of the sequences from Taiwan are likely caused by a mosaic *penA* allele, which arises from homologous recombination between related species such as *N. meningitidis*, *N. gonorrhoeae* (*27*), and other commensal *Neisseria* species. Despite the presence of common mutations between the 5 strains from Taiwan and the 5 strains from Fiji, the *penA* alleles from the strains from Fiji are in fact distinct. Of the strains from Fiji, 2 *penA* allele numbers were identified, 24 and ≈52 (Appendix 2 Table). Several strains that were isolated from Asia, New Zealand, Europe, and the United States have the *penA* allele number 24, along with 18 of the 23 strains from Fiji.

### Outbreak Response

A National Meningococcal Taskforce was established in Fiji in March 2018 to coordinate the outbreak response and focused on multiple action items. The action items were enhancing disease surveillance and case detection, early case management, contact tracing, and prevention. 

#### Enhanced Disease Surveillance and Case Detection

National surveillance was coordinated by the Fiji CDC by using laboratory and active surveillance (case investigation for suspected cases and contacts). Standardized data case investigation forms were created to enhance clinical and laboratory surveillance. Risk communications focused on educating the public to enable early recognition of symptoms and encourage seeking care early. Health education materials were created, and mass media outlets were used to raise public awareness. Large gatherings such as sporting events and schools were targeted for intensive risk communication activities. Awareness for clinicians was raised by using alert circulars, interim guidance, and training to enable early recognition of cases.

#### Early Case Management

The existing Public Health Management of Meningococcal Disease Guidelines were revised and made available to clinicians and public health teams during the week of outbreak declaration. Nationwide training on the new guidelines was conducted 2 weeks after outbreak declaration. Ceftriaxone was made available in all medical subdivisions because the MenC strain was resistant to penicillin, which was previously the first line treatment for suspected cases of meningococcal disease. Previously, ceftriaxone was only available in the 3 divisional hospitals.

#### Contact Tracing

Early in the outbreak, household and close contacts of IMD cases were given ciprofloxacin (500 mg in a single dose) for secondary prophylaxis. Once the MenC strain was identified as resistant to ciprofloxacin, the guidelines were revised to include rifampin (600 mg 1×/d for 2 days), and it was made available in all subdivisions.

#### Prevention

Risk communications focused on early recognition of symptoms and basic hygiene for prevention of transmission. Infection prevention control measures were strengthened among healthcare workers (HCW).

To control the first institutional outbreak at a boarding school, a vaccination campaign was conducted during July 25–27, 2017. A total of 587 students, teaching staff, and HCW were vaccinated with a quadrivalent (serogroup A, C, W, and Y) vaccine. No further cases of meningococcal disease were reported from the school following vaccination.

Subsequently, a nationwide targeted mass vaccination campaign among 1–19-year-old persons was conducted after the nationwide outbreak declaration. During May–October 2018, a total of 315,876 (91%) people received monovalent MenC vaccine (Fiji MoHMS, unpub. data). The vaccines were funded by the government of Fiji with assistance from the governments of Australia and New Zealand, World Health Organization, and United Nation Children’s Fund.

### Postoutbreak Surveillance

Public health surveillance of IMD continued under the VPD Unit in Fiji CDC. PCR was conducted on CSF samples at NPHL and in 2023 included serogroup determination. Selected samples were sent to MDU for serogroup determination. During January 1, 2019–August 30, 2023, a total of 23 confirmed cases of IMD were reported (Figure 2). In 2019, there were 2 clusters (April–May and August) of MenC in the Western division with an epidemiologic link identified through outbreak investigation (Figure 2). Serogroups in the period after the outbreak included 10 MenC (9 in 2019 and 1 in 2020), 8 MenB, and 1 MenY, whereas 4 IMD were confirmed by culture or direct antigen test with no serogroup information. Most (8/10) MenC cases were outside the mass vaccination age group (1 was in an 11-month-old baby and 7 were in adults, 22–67 years of age). The MenC strains detected during the postoutbreak period were MLST 4821. There were no MenC cases detected in 2021, 2022, or as of August 2023.

## Discussion

We have described an outbreak of IMD caused by a serogroup C MLST 4821 strain of *N. meningitidis* that is a novel occurrence of the disease in Fiji. IMD cases in Fiji were low and sporadic, and we could not obtain any report of serogroup C meningococcal disease in Fiji before 2016. Routine vaccination for IMD was not available, and therefore it can be expected that there was no herd immunity in the population at the time.

The first case of MenC identified was from an infant in the Western division in August 2016. Because of limited laboratory capacity at the subdivision level and low index of suspicion among HCW, MenC was likely underreported during the initial stages of the outbreak. The lack of capacity for serogrouping *N. meningitidis* in Fiji limited the ability to identify the presence of a vaccine amenable serogroup. The outbreak strain was resistant to ciprofloxacin and penicillin, which raised public health concerns because ciprofloxacin was the main antimicrobial drug for secondary prophylaxis and penicillin was the first-line treatment for suspected cases. Ciprofloxacin resistance was not identified until June 2017. Some disease transmission was likely because of the ineffective chemoprophylactic management of contacts early in the outbreak.

It is likely the serogroup C strain was imported by a returned traveler to Fiji because *N. meningitidis* serogroup C had not been reported in Fiji or the Pacific region before 2016. MenC MLST 4821 resistant to ciprofloxacin is an endemic strain in China (*28*–*30*), and imported cases have been reported in Canada (*31*) and Japan (*32*). When given global context, strains that were isolated from China within the MLST 4821 CC appear to represent a common ancestor of the strains from Fiji, supporting the hypothesis of the introduction of a single strain that was then transmitted locally. Importation of a contagion leading to an outbreak had previously been reported in Fiji when a measles outbreak occurred in 2006, which was traced to a tourist who visited the country (*33*).

A likely factor contributing to the outbreak was the category 5 tropical cyclone Winston, which hit Fiji in early 2016 and caused extensive damage to infrastructure, housing, and population displacement (*34*). The areas that suffered the greatest effects were the Western, Northern, and Eastern divisions (*35*). The 2 boarding schools where institutional outbreaks occurred sustained heavy damage to dormitories from the cyclone. That damage contributed to overcrowding, a well-described risk factor for spread of meningococcal disease. The first institutional outbreak coincided with a school holiday period and the exodus of students from school to surrounding areas for the holiday period that likely led to spread of MenC around the country in 2017.

The IMD outbreak affected the indigenous population to a much larger extent than other ethnic groups within the country. Disease transmission might have been affected by the social patterns of this ethnic group; living with extended families is common, large social gatherings are frequent, and population mobility between communities and islands is high. Disproportionately higher incidence of IMD has also been described in the indigenous populations of Australia (*36*) and New Zealand (*4*).

This outbreak demonstrated the importance of molecular diagnostics. Most CSF negative cultures tested positive by using CSF PCR at NPHL, which was the only medical laboratory conducting real-time PCR in Fiji at the time. In 2023, the capacity to serogroup samples was developed, which strengthened the surveillance capacity in Fiji. The early identification of *N. meningitidis* cases, including vaccine preventable serogroups, because of serogrouping availability is critical for early control of IMD.

In conclusion, an epidemic of IMD occurred in Fiji during 2017–2018, caused by a serogroup C MLST 4821 strain of *N. meningitidis* that was mostly likely introduced into the country by travel. The young and indigenous populations were disproportionately affected. Public health measures such as rapid case identification and management, contact tracing, prophylaxis for close contacts, public risk communications, HCW training and awareness, and targeted mass vaccination were effective in controlling the outbreak.

Appendix 1 Additional information about meningococcal C disease outbreak caused by multidrug resistant *Neisseria meningitidis*, Fiji.

Appendix 2 Additional information about characteristics of *Neisseria meningitidis* strains from Fiji and publicly available data.

## References

[1] Harrison LH, Trotter CL, Ramsay ME. Global epidemiology of meningococcal disease. Vaccine. 2009;27(Suppl 2):B51–63. 10.1016/j.vaccine.2009.04.063 19477562

[2] Jafri RZ, Ali A, Messonnier NE, Tevi-Benissan C, Durrheim D, Eskola J, et al. Global epidemiology of invasive meningococcal disease. Popul Health Metr. 2013;11:17. 10.1186/1478-7954-11-17 24016339 PMC3848799

[3] Jelfs J, Munro R. Epidemiology of meningococcal disease in Australia. J Paediatr Child Health. 2001;37(s5):S3–6. 10.1046/j.1440-1754.2001.00680.x 11885734

[4] Baker MG, Martin DR, Kieft CE, Lennon D. A 10-year serogroup B meningococcal disease epidemic in New Zealand: descriptive epidemiology, 1991-2000. J Paediatr Child Health. 2001;37(s5):S13–9. 10.1046/j.1440-1754.2001.00722.x 11885731

[5] Lawrence GL, Wang H, Lahra M, Booy R, McIntyre PB. Meningococcal disease epidemiology in Australia 10 years after implementation of a national conjugate meningococcal C immunization programme. Epidemiol Infect. 2016;144:2382–91. 10.1017/S0950268816000704 27094814 PMC9150535

[6] Lopez L, Sherwood J. The epidemiology of meningococcal disease in New Zealand in 2013. Wellington, New Zealand: Institute of Environmental Science and Research Ltd.; 2014 [cited 2022 Apr 15]. https://www.esr.cri.nz/media/wvgmuulc/esr-invasive-meningococcal-disease-annual-report-2013.pdf

[7] Fiji Islands Bureau of Statistics. Population and housing census 2017. 2018 [cited 2018 Apr 27]. https://www.statsfiji.gov.fj/index.php/2017_Population_and_Housing_Census_Release_1.pdf

[8] Fiji Ministry of Health and Medical Services. Annual report 2016. 2017 [cited 2022 April 15]. http://www.health.gov.fj/wp-content/uploads/2018/03/MoHMS-Jan-July-Report-2016.pdf

[9] Fiji Ministry of Health and Medical Services. Communicable diseases surveillance and outbreak management guidelines. 2016 [cited 2022 May 5]. https://www.health.gov.fj/wp-content/uploads/2018/08/Fiji-Communicable-Disease-Surveillance-and-Outbreak-Response-Guidelines-2016-1.pdf

[10] World Health Organization. Using Mobile Technology for Post-disaster Enhanced Surveillance in Fiji. 2016 [cited 2022 February 4]. http://www.wpro.who.int/southpacific/mediacentre/releases/2016/mobiletech_surveillance/en

[11] Biaukula VL, Tikoduadua L, Azzopardi K, Seduadua A, Temple B, Richmond P, et al. Meningitis in children in Fiji: etiology, epidemiology, and neurological sequelae. Int J Infect Dis. 2012;16:e289–95. 10.1016/j.ijid.2011.12.013 22342257

[12] Dunne EM, Mantanitobua S, Singh SP, Reyburn R, Tuivaga E, Rafai E, et al. Real-time qPCR improves meningitis pathogen detection in invasive bacterial-vaccine preventable disease surveillance in Fiji. Sci Rep. 2016;6:39784. 10.1038/srep39784 28009001 PMC5180226

[13] World Health Organization. Recommended surveillance standards. Second edition. 1999 [cited 2017 March 4]. https://cdn.who.int/media/docs/default-source/documents/publications/who-recommended-surveillance-standards17363eff-9860-48c1-9f5f-3c0c3a4f955d.pdf

[14] Center for Disease Control and Prevention. Meningococcal disease (*Neisseria meningitidis*) 2015 case definition. 2015 [cited 2022 Sep 7]. https://ndc.services.cdc.gov/case-definitions/meningococcal-disease-2015

[15] Clinical and Laboratory Standards Institute. Performance standards for antimicrobial susceptibility testing; twenty-seventh informational supplement (M100-S27). Wayne (PA): The Institute; 2017.

[16] Vuong J, Collard JM, Whaley MJ, Bassira I, Seidou I, Diarra S, et al. Development of real-time PCR methods for the detection of bacterial meningitis pathogens without DNA extraction. PLoS One. 2016;11:e0147765. 10.1371/journal.pone.0147765 26829233 PMC4735509

[17] Prjibelski A, Antipov D, Meleshko D, Lapidus A, Korobeynikov A. Using SPAdes de novo assembler. Curr Protoc Bioinformatics. 2020;70:e102. 10.1002/cpbi.102 32559359

[18] Sherry NL, Horan KA, Ballard SA, Gonҫalves da Silva A, Gorrie CL, Schultz MB, et al. An ISO-certified genomics workflow for identification and surveillance of antimicrobial resistance. Nat Commun. 2023;14:60. 10.1038/s41467-022-35713-4 36599823 PMC9813266

[19] Katz LS, Griswold T, Morrison SS, Caravas JA, Zhang S, den Bakker HC, et al. Mashtree: a rapid comparison of whole genome sequence files. J Open Source Softw. 2019;4:1762. 10.21105/joss.01762 35978566 PMC9380445

[20] Guangchuang Y. David Smith, Huachen Zhu, Yi Guan, Tommy Tsan-Yuk Lam. ggtree: an R package for visualization and annotation of phylogenetic trees with their covariates and other associated data. Methods Ecol Evol. 2017;8:28–36. 10.1111/2041-210X.12628

[21] Wickham H. ggplot2: Elegant Graphics for Data Analysis, 3rd edition. New York: Springer-Verlag. 2016.

[22] Revell LJ. phytools: An R package for phylogenetic comparative biology (and other things). Methods Ecol Evol. 2012;2012:217–23. 10.1111/j.2041-210X.2011.00169.x

[23] Fiji Ministry of Health and Medical Services. Annual report 2015. 2016 [cited 2023 Nov 22]. http://www.health.gov.fj/wp-content/uploads/2018/03/MoHMS-Jan-July-Report-2016.pdf

[24] Fiji Ministry of Health and Medical Services. Media release: meningococcal disease outbreak. 2017 [cited 2022 Apr 22]. http://www.health.gov.fj/media-release-meningococcal-disease-outbreak

[25] Fiji Sun. Ministry of Health has lifted meningococcal C threats. 2018 [cited 2022 Apr 4]. https://fijisun.com.fj/2018/12/19/ministry-of-health-has-has-lifted-meningococcal-c-threats

[26] Jolley KA, Bray JE, Maiden MCJ. Open-access bacterial population genomics: BIGSdb software, the PubMLST.org website and their applications. Wellcome Open Res. 2018;3:124. 10.12688/wellcomeopenres.14826.1 30345391 PMC6192448

[27] Zapun A, Morlot C, Taha MK. Resistance to β-lactams in *Neisseria* ssp due to chromosomally encoded penicillin-binding proteins. Antibiotics (Basel). 2016;5:35. 10.3390/antibiotics5040035 27690121 PMC5187516

[28] Zhou H, Gao Y, Xu L, Li M, Li Q, Li Y, et al. Distribution of serogroups and sequence types in disease-associated and carrier strains of *Neisseria meningitidis* isolated in China between 2003 and 2008. Epidemiol Infect. 2012;140:1296–303. 10.1017/S0950268811001865 21929839

[29] Guo Q, Mustapha MM, Chen M, Qu D, Zhang X, Chen M, et al. Evolution of sequence type 4821 clonal complex meningococcal strains in China from prequinolone to quinolone era, 1972–2013. Emerg Infect Dis. 2018;24:683–90. 10.3201/eid2404.171744 29553310 PMC5875256

[30] Chen M, Harrison OB, Bratcher HB, Bo Z, Jolley KA, Rodrigues CMC, et al. Evolution of sequence type 4821 clonal complex hyperinvasive and quinolone-resistant meningococci. Emerg Infect Dis. 2021;27:1110–22. 10.3201/eid2704.203612 33754991 PMC8007298

[31] Tsang RS, Law DK, Deng S, Hoang L. Ciprofloxacin-resistant *Neisseria meningitidis* in Canada: likely imported strains. Can J Microbiol. 2017;63:265–8. 10.1139/cjm-2016-0716 28140652

[32] Kawasaki Y, Matsubara K, Takahashi H, Morita M, Ohnishi M, Hori M, et al. Invasive meningococcal disease due to ciprofloxacin-resistant *Neisseria meningitidis* sequence type 4821: The first case in Japan. J Infect Chemother. 2018;24:305–8. 10.1016/j.jiac.2017.11.001 29233459

[33] World Health Organization. Measles outbreak in Fiji, February–May 2006 Measles bulletin. 2006 [cited 2022 Apr 20] https://iris.who.int/bitstream/handle/10665/233188/WER8136_341-346.pdf

[34] Fiji Ministry of Health and Medical Services. Rapid public health risk assessment tropical cyclone Winston republic of Fiji. 2016 [cited 2022 Apr 4]. http://www.health.gov.fj/wp-content/uploads/2016/03/20160315-Rapid-Health-Risk-Assessment-TC-Winston-Mar2016-for-editing_14-March-2016_final-2.pdf

[35] Government of Fiji. Post disaster needs assessment, tropical cyclone Winston, Fiji. 2016 [cited 2022 Apr 15]. https://www.gfdrr.org/sites/default/files/publication/Post%20Disaster%20Needs%20Assessments%20CYCLONE%20WINSTON%20Fiji%202016%20(Online%20Version).pdf

[36] Patel MS, Merianos A, Hanna JN, Vartto K, Tait P, Morey F, et al. Epidemic meningococcal meningitis in central Australia, 1987-1991. Med J Aust. 1993;158:336–40. 10.5694/j.1326-5377.1993.tb121793.x 7605395

