# Supplementary material for: Meningococcal C Disease Outbreak Caused by Multidrug-Resistant Neisseria meningitidis, Fiji
Source: Emerg Infect Dis. 2025 Jan;31(1):32–40. doi: 10.3201/eid3101.240476 (PMC11682797; doi:10.3201/eid3101.240476)
Supplement: Appendix 1 — Additional information about meningococcal C disease outbreak caused by multidrug resistant Neisseria meningitidis, Fiji. [file 24-0476-Techapp-s1.pdf]

Article DOI: https://doi.org/10.3201/eid3101.240476

EID cannot ensure accessibility for supplementary materials supplied by authors. Readers who have difficulty accessing supplementary content should contact the authors for assistance.

Meningococcal C Disease Outbreak Caused by Multidrug-Resistant *Neisseria meningitidis*, Fiji

Appendix 1

Appendix 1 Table 1. Sequences and concentrations of the primers and probes used in Fiji Centre of Disease Control

Target gene	Forward primer (F)	Reverse primer (R)	Probe (P)	Concentration of oligo (F/R/P) per reaction (nM)
sodC	GCACACTTAGGTGAT TTACCTGCAT	CCACCCGTGTGGAT CATAATAGA	FAM-CATGATGGCACAGCAACAAATCCTG TTT-BHQ1	300/600/100
sacB	AAAATTCAATGGGTA TATCACGAAGA	ATATGGTGCAAGCT GGTTTCAATAG	FAM-CTA AAA G"T"A GGA AGG GCA CTT TGT GGC ATA AT-SpC6	
synD	GCT ACC CCA TTT CAG ATG ATT TGT	ACCAGCCGAGGGT TTATTTCTAC	FAM-AAG AGA TGG GYA ACA AC"T" ATG TAA TGT CTT TAT TT-SpC6	
synE	CCC TGA GTA TGC GAA AAA AAT T	TGCTAATCCCGCCT GAATG	FAM-TTT CAA TGC "T"AA TGA ATA CCA CCG TTT TTT TGC-SpC6	
synG	TATTTATGGAAGGCA TGGTGTATG	TTGCCATTCCAGAA ATATCACC	FAM-AAA TAT GGA GCG AAT GAT TAC AGT AAC TAT AAT GAA-SpC6	
xcbB	TGTCCCCAACCGTTT ATTGG	TGCTGCTATCATAG CCGCC	FAM-TGT TTG CCC ACA TGA ATG GCG G-BHQ1	
synF	TCCGAGCAGGAAAT TTATGAGAATAC	TTGCTAAAATCATTC GCTCCATAT	FAM-TAT GGT G"T"A CGA TAT CCC TAT CCT TGC CTA TAA T-SpC6	

Appendix 1 Table 2. Clinical features and admission diagnoses of confirmed and probable cases of IMD in Fiji, 2016-2018

Characteristics	Total(n=90*) no. (%)	<5 years(n=29) no. (%)	≥ 5 years(n=61) no. (%)
**Symptoms**			
Fever	88(97.8)	28 (96.6)	60(98.4)
Vomiting	55(61.1)	12(41.4)	43(70.5)
Headache	43(47.8)	2(6.9)	41(67.2)
Skin rashes	28(31.1)	10(37.9)	17 (27.9)
Loss of appetite	25(27.8)	13(44.8)	12(19.7)
Neck stiffness	24(26.7)	5(17.2)	19(31.1)
Confusion	20(22.2)	3(10.3)	17(27.9)
Seizure	19 (21.1)	8(27.6)	11(18.0)
Nausea	17 (18.9)	3(10.3)	14 (23.0)
Ecchymosis	7(7.8)	3(10.3)	4(6.4)
Photophobia	4(4.4)	0	4(6.6)
**Admission diagnoses**			
Meningitis	52 (57.8)	16 (55.2)	36 (59.0)
Meningococcaemia	16 (17.8)	6 (20.7)	10 (16.4)
Pneumonia	2 (2.2)	1 (3.4)	1 (1.6)
Other†	11(12.2)	4 (13.8)	7 (11.5)
Unknown	9 (10.0)	2 (6.9)	7 (11.5)

*Six patients with no clinical information were excluded

†Includes acute confusional state (n=3), acute gastroenteritis (n=3), contacts of meningococcaemia case (n=2), acute febrile illness (n=2) and septicaemia (n=1)

Appendix 1 Table 3. Antimicrobial susceptibility testing results performed in Fijian laboratories, 2016–2018*

Antimicrobials	Total	Resistant n	Susceptible n	Susceptible %
**All**				
Penicillin	42	27	15	36
Chloramphenicol	17	0	17	100
Ciprofloxacin	39	9	30	77
Erythromycin	19	1	18	95
Doxycycline	20	2	18	90
Rifampicin	15	0	15	100
Ceftriaxone	37	0	37	100
**Serogroup B**				
Penicillin	16	13	3	19
Chloramphenicol	6	0	6	100
Ciprofloxacin	16	1	15	94
Erythromycin	7	1	6	86
Doxycycline	8	2	6	75
Rifampicin	5	0	5	100
Ceftriaxone	15	0	15	100
**Serogroup C**				
Penicillin	24	13	11	46
Chloramphenicol	11	0	11	100
Ciprofloxacin	21	7	14	67
Erythromycin	11	0	11	100
Doxycycline	11	0	11	100
Rifampicin	10	0	10	100
Ceftriaxone	20	0	20	100

*Include all isolates (N=42), serogroup B (n=16), serogroup C (n=24) serogroup W (n=1) and unknown serogroup (n=1).

Appendix 1 Table 4. Antimicrobial susceptibility testing results performed in MDU laboratory on 15 serogroup C strains, 2016–2018*

Antimicrobials	Total	Resistant n†	Susceptible n	Susceptible %
Penicillin	15	14	1	7
Chloramphenicol	Not done	Not done	Not done	Not done
Ciprofloxacin	15	15	0	0
Erythromycin	Not done	Not done	Not done	Not done
Doxycycline	Not done	Not done	Not done	Not done
Rifampicin	14	1	13	93
Ceftriaxone	15	0	15	100

*MDU, Microbiological Diagnostic Unit
†There were 5 discordant AST results for ciprofloxacin and one for rifampicin where resistant strains in MDU were reported as susceptible (sensitive) in Fijian laboratories.

Appendix 1 Table 5. Characteristics of clonal cluster 4821 included in the whole genome sequencing analysis*

Isolate ID	Year of collection	FetA	PorA	fHbp	NHBA	NadA	BAST
AUSMDU00008749	2017	F3-3	5,2	215	503	0	NA
AUSMDU00008750	2017	F3-3	5,2	420	503	0	NA
AUSMDU00009809	2017	F3-3	5,2	22	503	0	3041
AUSMDU00009847	2017	F3-3	5,2	420	503	0	NA
AUSMDU00009811	2017	F3-3	5,2	22	503	0	3041
AUSMDU00013127	2017	F3-3	5,2	54	503	0	NA
AUSMDU00013128	2017	F3-3	5,2	420	503	0	NA
AUSMDU00013129	2017	NA	5,2	22	503	0	3041
AUSMDU00013130	2017	NA	5,2	90	503	0	NA
AUSMDU00013131	2017	F3-3	5,2	22	503	0	3041
AUSMDU00013132	2018	NA	5,2	13	503	0	NA
AUSMDU00013135	2018	F3-3	5,2	22	503	0	3041
AUSMDU00015102	2017	F3-3	5,2	22	503	0	3041
AUSMDU00015103	2017	F3-3	5,2	22	503	0	3041
AUSMDU00015105	2018	F3-3	5,2	420	503	0	NA
AUSMDU00016639	2018	NA	5,2	22	503	0	3041
AUSMDU00018457	2018	F3-3	5,2	22	503	0	3041
AUSMDU00018459	2018	F3-3	5,2	22	503	0	3041
AUSMDU00036081	2019	F3-3	5,2	393	503	0	NA
AUSMDU00036082	2019	F3-3	5,2	new	503	0	NA
AUSMDU00036083	2019	F3-3	5,2	new	503	0	NA
AUSMDU00036084	2019	F3-3	5,2	new	503	0	NA

Isolate ID	Year of collection	FetA	PorA	fHbp	NHBA	NadA	BAST
AUSMDU00036089	2019	F3-3	5,2	new	503	0	NA
ERR3336532	2014	new	20,23-7	141	669	0	NA
SRR10610911	2018	F3-3	5,2	22	503	0	3041
SRR18091102	2014	F5-8	12-14,new	404	697	0	NA
SRR18091103	2013	F5-8	12-14,13-20	404	697	0	3729
SRR18091110	2012	F3-3	7-2,14	80	503	0	802
SRR18091130	2020	F3-3	20,23	22	503	0	4460
SRR18091183	2018	F3-3	20-3,23-6	420	503	0	4478
SRR18091189	2011	F3-3	20,23-1	22	503	0	3703
SRR18091213	2017	F3-3	20-3,23-6	420	503	0	4478
SRR18091214	2017	F3-3	20-3,23-6	420	503	0	4478
SRR18091215	2017	F3-3	20-3,23-6	420	503	0	4478
SRR18091216	2017	F3-3	20-3,23-6	420	503	0	4478
SRR18091223	2016	F3-3	20,23-7	22	503	0	4492
SRR18091240	2008	F5-8	12-14,13-20	404	697	0	3729
SRR18091246	2007	F3-36	20,23	488	669	0	4497
SRR18091255	2007	F5-8	12-14,13-20	404	697	0	3729

*BAS, Bexsero antigen sequence type; ID, identification.

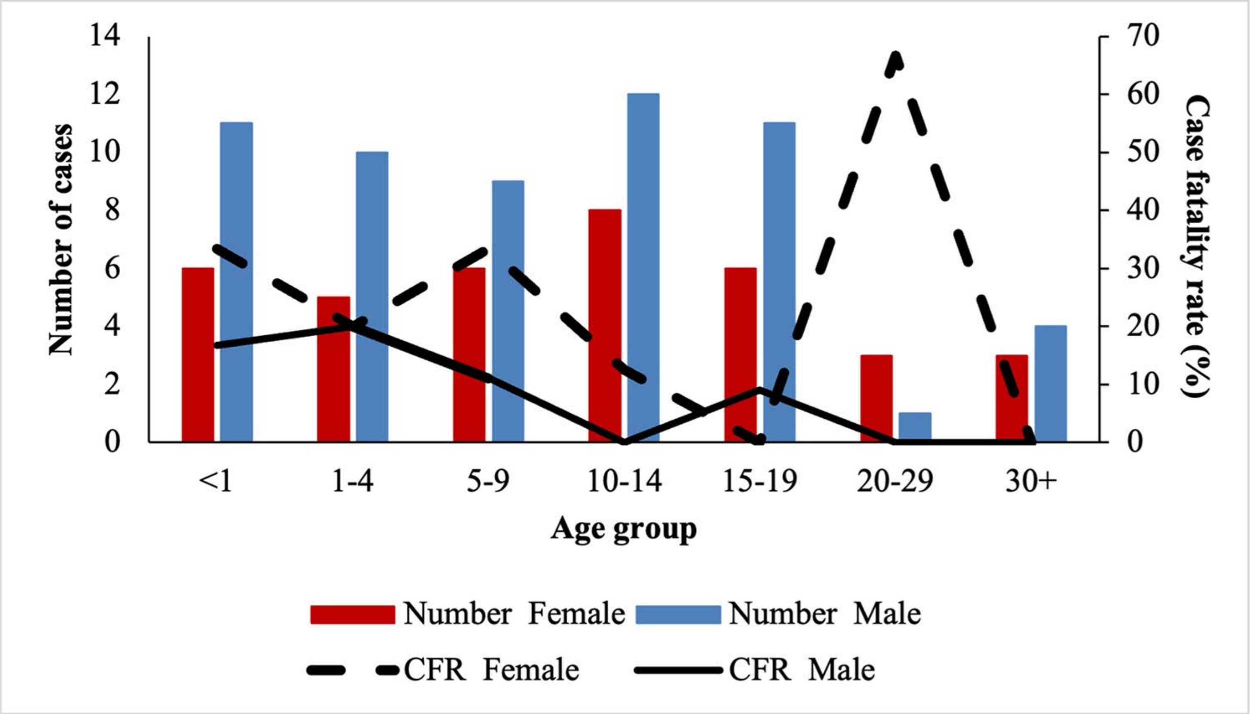

Appendix 1 Figure. Confirmed and probable IMD cases and case fatality rate in Fiji by age group and sex, 2016–2018
